# The Therapeutic Environment in Psychiatric Residential Rehabilitation: A Key Factor in Emotional Dysregulation Management

**DOI:** 10.1192/j.eurpsy.2026.11701

**Published:** 2026-07-31

**Authors:** M. V. Mino’, A. Vacca, F. Franza, A. Litta

**Affiliations:** 1 Psychiatric Rehabilitation Center “Don Tonino Bello”- Assoc. M.I.T.A.G., Brindisi; 2 Mental Health Department, ASL Taranto, Taranto; 3 Psychiatric Studies Center" (Cen.Stu.Psi.), Provaglio d’Iseo (BS); 4 Department of Precision and Regenerative Medicine and Ionian Area (DiMePRe-J), University of Bari “Aldo Moro”, Bari, Italy

## Abstract

**Introduction:**

Emotional dysregulation is a core feature of several psychiatric disorders, particularly borderline personality disorder, bipolar disorder, PTSD, and neurodevelopmental conditions. In residential settings, the therapeutic environment actively influences patients’ emotional regulation

**Objectives:**

To analyse the role of the therapeutic environment in psychiatric residential rehabilitation, highlighting its impact on emotional dysregulation.

**Methods:**

A **narrative review** of empirical studies and selected systematic reviews (Delaney & Johnson 2006; McAndrew et al. 2022; Zadeh et al. 2024) was conducted. The analysis focused on architectural design, relational dynamics, and organizational factors influencing therapeutic milieus in psychiatric residential rehabilitation

**Results:**

Findings indicate that structured therapeutic environments can significantly reduce aggression, stress, and acting-out behaviours. Elements such as natural light, access to green spaces, noise reduction, and the availability of personalizable areas contribute to improved mood and treatment adherence. Aesthetic and acoustic design not only benefits patients but also supports staff well-being, fostering more effective co-regulation. Evidence shows that environmental modifications may lead to over 30% fewer aggressive incidents.

**Conclusions:**

Overall, the therapeutic environment emerges as an active agent in psychiatric care, crucial for modulating emotional dysregulation. Integrating architectural design, sensory stimuli, and relational aspects into rehabilitation pathways appears to enhance clinical outcomes, although further research is needed to validate tools capable of objectively measuring environmental effects on emotional regulation.

**Disclosure of Interest:**

None Declared

